# Clinical characteristics, treatment, and blood pressure control in patients with hypertension seen by primary care physicians in Spain: the IBERICAN study

**DOI:** 10.3389/fcvm.2023.1295174

**Published:** 2023-12-20

**Authors:** Miguel A. Prieto-Díaz, Vicente Pallares-Carratala, Rafael Manuel Micó-Pérez, Carlos Escobar-Cervantes, Vicente Martín-Sanchez, Antonio Coca, Alfonso Barquilla-García, Sonsoles M. Velilla-Zancada, José Polo-García, Antonio Segura-Fragoso, Leovigildo Ginel-Mendoza, Álvaro Hermida-Ameijerias, Sergio Cinza-Sanjurjo, the Investigators of the IBERICAN study and of the Spanish Society of Primary Care Physicians SEMERGEN Foundation

**Affiliations:** ^1^ Vallobín-La Florida Health Center, Principality of Asturias Health Service, Oviedo, Spain; ^2^ Faculty of Medicine, University of Santiago de Compostela, A Coruña, Spain; ^3^ Health Surveillance Unit, Mutual Insurance Union, Castellon, Spain; ^4^ Department of Medicine, Jaume I University, Castellon, Spain; ^5^ Xàtiva–Ontinyent Department of Health, Fontanars Dels Alforins Health Center, Valencia, Spain; ^6^ Cardiology Service, La Paz University Hospital, Madrid, Spain; ^7^ Institute of Biomedicine (IBIOMED), Epidemiology and Public Health Networking Biomedical Research Centre (CIBERESP), University of León, León, Spain; ^8^ Hypertension and Vascular Risk Unit, Department of Internal Medicine, Hospital Clínic, University of Barcelona, Barcelona, Spain; ^9^ Trujillo Health Center, Extremadura Health Service, Cáceres, Spain; ^10^ Joaquin Elizalde Health Center, Rioja Health Service, Logroño, Spain; ^11^ Casar de Cáceres Health Center, Extremadura Health Service, Cáceres, Spain; ^12^ Epidemiology Unit, Semergen Research Agency, Madrid, Spain; ^13^ Ciudad Jardín Health Center, Andalusia Health Service, Málaga, Spain; ^14^ Department of Internal Medicine, University Hospital of Santiago de Compostela, Coruña, Spain; ^15^ Milladoiro Health Centre, Health Area of Santiago de Compostela, Health Research Institute of Santiago de Compostela (IDIS), A Coruña, Spain; ^16^ Networking Biomedical Research, Centre-Cardiovascular Diseases (CIBERCV), Santiago de Compostela, Spain

**Keywords:** blood pressure, combined therapy, hypertension, primary care, cardiovascular risk

## Abstract

**Objectives:**

To determine the clinical profile, according to the history of hypertension, the risk of developing hypertension, current antihypertensive treatment and BP control rates in patients with hypertension from the IBERICAN cohort.

**Methods:**

IBERICAN is an ongoing prospective cohort study, whose primary objective is to determine the frequency, incidence, and distribution of CVRF in the adult Spanish population seen in primary care settings. This analysis shows the baseline clinical characteristics of patients with hypertension. Adequate BP control was defined as BP <140/90 mmHg according to 2013 ESH/ESC guidelines.

**Results:**

A total of 8,066 patients were consecutively included, of whom 3,860 (48.0%) had hypertension. These patients were older (65.8 ± 10.9 vs. 51.6 ± 14.7 years; *p* < 0.001), had more cardiovascular risk factors, target organ damage and cardiovascular disease (CVD) in comparison with those without hypertension. The risk of hypertension increased with the presence of associated CV risk factors and comorbidities, particularly diabetes, obesity and the metabolic syndrome, and decreased with the intensity of physical activity. Regarding antihypertensive treatments, 6.1% of patients did not take any medication, 38.8% were taking one antihypertensive drug, 35.5% two drugs, and 19.6% three or more antihypertensive drugs. Overall, 58.3% achieved BP goals <140/90 mmHg. A greater probability of BP control was observed with increasing age of patients and the greater number of antihypertensive drugs. Blood pressure control was lower in hypertensive patients with diabetes, obesity, the metabolic syndrome, increased urinary albumin excretion, higher pulse pressure, and lack of antihypertensive treatment.

**Conclusions:**

About half of patients attended in primary care settings have hypertension in Spain. Patients with hypertension have a worse CV clinical profile than non-hypertensive patients, with greater association of CVRF and CVD. Around four out of ten patients do not achieve the recommended BP goals, and higher use of combination therapies is associated with a better BP control.

## Introduction

Hypertension is the first cause of death and disability worldwide and one of the main modifiable risk factors for the development of cardiovascular disease (CVD) (1). Unfortunately, despite the efforts performed in the last decades to reduce hypertension burden through the reduction of blood pressure (BP) with antihypertensive treatment (2, 3), the fact is that awareness, treatment, and BP control remains suboptimal worldwide (4, 5). Despite the improvement in the prevention and treatment of CVD in Spain, it still remains the first cause of death, accounting for one quarter of all deaths in the country (6, 7). In fact, compared to 2021, death from cardiovascular (CV) causes only descended by 0.6% in 2022 in Spain (8).

Population-based studies in Spain have shown that around one third of adults have hypertension (more than 60% in those >65 years) (9, 10), but in subjects attended in primary care, these numbers reach to 50%–60% of patients (6, 11). Previous studies have shown that only half of treated hypertensive patients are adequately controlled, which would translate into around 30,000 CV deaths annually attributable to hypertension (7). Therefore, an improvement of the awareness, treatment and BP control in our country is urgently needed. In this context, monitoring the evolution of the proportion of patients with hypertension, as well as the optimal therapeutic approach of the hypertensive population seems mandatory (7). Although previous studies have analyzed these critical points (11–14), it seems desirable an update of the current management and control of these patients. On the other hand, primary care is the best setting for promoting the management, follow-up and control of cardiovascular risk factors (CVRF), including hypertension (6, 15).

Although numerous previous studies have analyzed the clinical characteristics and management of patients with arterial hypertension, it is necessary to update these results in a contemporary cohort of patients, analyzing the impact of the latest arterial hypertension guidelines and modern therapies on the management and achievement of BP goals, as well as those factors associated with blood pressure control. Additionally, it is also important to determine whether predictors of developing hypertension have changed over time.

The IBERICAN study (Identification of Spanish Population in Cardiovascular and Renal Risk) is an ongoing prospective cohort study, whose primary aim is to determine the frequency, incidence, and geographical distribution of CVRF in adults attended in primary care in Spain (6, 16, 17). In this article we report the clinical profile according to the history of hypertension, the risk of developing hypertension, current antihypertensive treatment and BP control rates in patients with hypertension from the IBERICAN cohort.

## Methods

The design and characteristics of the study have been previously published (6, 16, 17). The IBERICAN study is an epidemiological, multicenter, observational, cohort study with a planned follow-up of 10 years, in which 8,066 patients between 18 and 85 years old were recruited by 519 primary care physicians, of the Spanish National Health System, between April 2014 and October 2018, by consecutive non-probabilistic sampling.

To estimate the sample size (18, 19), we followed the next steps: (1) we developed a multivariate Cox regression analysis predictive model to explain the effect of 10 independent variables on the incidence of cardiovascular events are, as the primary strictest objective of the IBERICAN study; (2) a minimum of 10–15 events in each variable was considered to estimate the number of needed events; (3) based on reported Spanish population data, the incidence of events in our population was estimated as 4.75/1,000 habitants/year; and (4) on the basis of this consideration, we calculated a sample size of 4.200–6.300 patients to predict 10–15 events in each variable. A 10% possible loose of patients along the follow-up was added to this figure, so the final number increased to a size of 6,600 patients. When the recruitment period was finished 8,066 patients had completed valid data.

To answer the main objective of the study a double analysis was planned: a longitudinal design to answer questions about the incidence of CVRF and CVD; and a cross-sectional design planned to answer questions about prevalence. This manuscript include data of this later analysis planned to know the prevalence of hypertension and its association with the other associated variables.

All patients provided written informed consent before inclusion. The study was approved by the Clinical Research Ethics Committee of the Hospital Clínico San Carlos in Madrid on 21 February 2013 (C.P. IBERICAN-C.I. 13/047-E) and is registered at https://clinicaltrials.gov with the number NCT02261441.

Patients included in the study underwent a conventional examination of clinical parameters and were treated according to usual clinical practice. As a result, non-pharmacological and pharmacological recommendations were performed according to physicianś judgement. No specific recommendations were performed for being included in the study. At baseline, socio-demographic data, CVRF (age, hypertension, obesity, smoking, diabetes mellitus, hypercholesterolemia, and sedentary lifestyle), HMOD, [high pulse pressure, ankle-brachial index <0.9, electrocardiographic or echocardiographic left ventricular hypertrophy (LVH), and urinary albumin excretion] and established CVD (ischemic heart disease, stroke, peripheral artery disease, heart failure, retinopathy, chronic kidney disease and atrial fibrillation) were collected. The variables were defined in accordance with the 2013 guidelines of the European Societies of Hypertension and Cardiology (ESH/ESC) (20), the current guidelines at the moment of inclusion. Obesity was defined as a body mass index >30 kg/m^2^, and sedentarism as doing physical less than 30 min of physical activity or absence of activity. The serum biochemistry data were recorded from the lab tests performed in the local laboratories within the last six months previous to the inclusion. The CV risk stratification of patients was carried out following the SCORE tables for low-risk countries, as this stratification was recommended at the time of enrolling (20). Patients outside the age limits for the SCORE algorithm were considered as the age closest to the corresponding interval. Data were collected in an electronic case report forms (eCRF) specifically developed for the study.

Subjects were defined as having hypertension if they had been already diagnosed as such by their physicians, or those who were taking antihypertensive medication.

According to the 2013 ESH/ESC guidelines (20), blood pressure measurement was performed with the patient seated, after 5 min of rest, through 2 determinations and obtaining the average. Adequate BP control was defined as SBP <140 mmHg, in all patients, except in individuals older than 80 years, in which a SBP between 150 and 140 mmHg was considered. Regarding DBP, the target <90 mmHg was considered in all patients, except in patients with diabetes in whom the DBP target was <85 mmHg. The BP control criteria are also in accordance with the current guidelines ESH 2023 (21). Treatments prescribed by physicians at baseline were recorded. The number and type of antihypertensive drugs [including angiotensin-converting enzyme inhibitors (ACEi), angiotensin receptor blockers (ARB), diuretics, calcium channel blockers (CCB), beta blockers, alpha blockers, renin inhibitors, and central acting agents] were also recorded in the overall hypertensive population.

## Statistical analysis

Qualitative variables were expressed as frequencies and percentages and quantitative variables as the mean and standard deviation (SD). For the comparison of subgroups of patients (hypertension vs. no hypertension and men vs. women), parametric tests (Student's *t*-test) or non-parametric tests (Mann–Whitney *U*-test) were used for quantitative variables, according to the specific characteristics of the variables examined. The Chi-square test was used for qualitative variables.

In order to evaluate which factors were associated with the presence of hypertension and also those variables that were independently associated with BP control, binary logistic regression models were built. The multivariate models begun to be constructed by introducing those factors with a significance of *p* < 0.150 in the bivariates by the automatic variable selection method by steps backward. Only the significant factors were finally considered to build the model. Adjusted odds ratios (OR) and their 95% confidence interval were presented in both cases. In all contrasts, the null hypothesis was rejected when the alpha error was lower than 0.05. The statistical package SPSS 23.0 (Statistical Package for Social Sciences) for Windows (Armonk, NY, USA: IBM Corp. Released 2013. IBM SPSS Statistics for Windows, version 23.0 Armonk, NY: IBM Corp.) was used for the data analysis.

## Results

A total of 519 primary care physicians recruited 8,066 patients, of whom 3,860 (48.0%) had hypertension. Compared with patients without hypertension, patients with hypertension were older (65.8 ± 10.9 vs. 51.5 ± 14.6 years; *p* < 0.001), more frequently men (50.5% vs. 49.5%; *p* < 0.001), had a lower level of education (13.8% vs. 4.7% without any formal study; *p* < 0.001) and a higher body mass index (BMI 30.06 ± 5.5 vs. 27.03 ± 4.9 kg/m^2^; *p* < 0.001). Fasting glucose, triglycerides, creatinine, albumin-to-creatinine ratio and uric acid levels were also higher among patients with hypertension, whereas HDL-cholesterol and estimated glomerular filtration rate (eGFR) were lower (Table 1).

**Table 1:** Clinical characteristics of the study population according to the history of hypertension (*n* = 8,066).

	No hypertension (*n* = 4.190; 52.0%)	Hypertension (*n* = 3.860; 48.0%)	*p*
Biodemographic data			
Age, years	51.5 ± 14.6	65.8 ± 10.9	<0.001
<40 years, *n* (%)	957 (22.9)	58 (1.5)	<0.001
40–49 years, *n* (%)	890 (21.3)	273 (7.1)	
50–59 years, *n* (%)	1,093 (26.1)	793 (20.5)	
60–69 years, *n* (%)	787 (18.8)	1,205 (31.2)	
70–79 years, *n* (%)	370 (8.8)	1,184 (30.7)	
≥80 years, *n* (%)	89 (2.1)	348 (9.0)	
Sex, female, *n* (%)	2,478 (59.1)	1,913 (49.5)	<0.001
Race, *n* (%)			
Caucasian	3,972 (94.8)	3,765 (97.5)	<0.001
Black	22 (0.5)	19 (0.5)	
Latin	156 (3.7)	65 (1.7)	
Asian	6 (0.1)	3 (0.1)	
Berber	34 (0.8)	9 (0.2)	
Habitat, *n* (%)			
Urban (>20,000 inhabitants)	2,583 (61.7)	2,256 (58.5)	0.013
Semi-urban (5,000–20,000 inhabitants)	867 (20.7)	863 (22.4)	
Rural (<5,000 inhabitants)	735 (17.6)	739 (19.1)	
Level of education, *n* (%)			
None	199 (4.7)	531 (13.7)	<0.001
Primary	2,045 (48.8)	2,346 (60.8)	
Secondary	1,154 (27.5)	686 (17.8)	
University	792 (18.9)	298 (7.7)	
Employment status, *n* (%)			
Works	2,376 (57.1)	1,026 (26.7)	<0.001
Unemployed	443 (10.6)	213 (5.5)	
Retired	835 (20.1)	2,023 (52.6)	
Student	100 (2.4)	6 (0.2)	
Housework	410 (9.8)	576 (15.0)	
Excessive alcohol consumption, *n* (%)	485 (11.6)	549 (14.3)	0.003
Physical examination			
Body mass index, kg/m^2^	27.03 ± 4.9	30.06 ± 5.5	<0.001
Systolic blood pressure, mmHg	123.3 ± 14.4	135.2 ± 15.3	<0.001
Diastolic blood pressure, mmHg	74.9 ± 9.7	78.6 ± 10.3	<0.001
Pulse pressure, mmHg	48.5 ± 11.4	56.6 ± 13.2	<0.001
Heart rate, bpm	72.8 ± 10.4	73.9 ± 11.3	<0.001
Biochemical parameters			
Fasting glucose, mg/dl	95.5 ± 24.1	109.7 ± 31.3	<0.001
HbA1c, % (among diabetics)	7.1 ± 1.4	7.0 ± 1.1	0.66
Total cholesterol, mg/dl	198.9 ± 38.6	191.0 ± 40.5	<0.001
HDL cholesterol, mg/dl	57.1 ± 15.5	52.6 ± 14.9	<0.001
LDL cholesterol, mg/dl	121.3 ± 34.6	113.4 ± 35.9	<0.001
Triglycerides, mg/dl	115.3 ± 80.2	135.4 ± 82.0	<0.001
Creatinine, mg/dl	0.83 ± 0.4	0.92 ± 0.5	<0.001
eGFR, ml/min/1.73 m^2^ (MDRD)	91.0 ± 23.9	80.4 ± 22.7	<0.001
eGFR, ml/min/1.73 m^2^ (CKD-EPI)	94.7 ± 18.5	80.6 ± 19.0	<0.001
Albumin-to-creatinine ratio, mg/g	1.05 ± 0.2	1.14 ± 0.4	<0.001
Uric acid, mg/dl	4.9 ± 1.4	5.6 ± 1.5	<0.001

eGFR, estimated glomerular filtration rate.

Cardiovascular risk factors and comorbidities were more common in the hypertensive population, including hypercholesterolemia (65.9% vs. 36.0%; *p* < 0.001), diabetes (31.6% vs. 9.6%; *P* < 0.001), obesity (45.9% vs. 22.6%; *p* < 0.001), and sedentary lifestyle (34.9% vs. 24.3%; *p* < 0.001). However, patients without hypertension were more commonly current smokers (21.2% vs. 13.7%; *p* < 0.001). As expected, the presence of any HMOD was also more frequent in patients with hypertension (44.3% vs. 14.7%; *p* < 0.001), as well as the presence of clinical CVD (24.1% vs. 9.3%; *p* < 0.001) (Table 2).

**Table 2:** Cardiovascular risk factors, hypertension-mediated organ damage and cardiovascular disease according to the history of hypertension (*n* = 8,066).

	No hypertension (*n* = 4,190; 52.0%)	Hypertension (*n* = 3,860 48.0%)	*p*
Cardiovascular risk factors			
Hypercholesterolemia, *n* (%)	1,509 (36.0)	2,541 (65.9)	<0.001
Diabetes, *n* (%)	404 (9.6)	1,219 (31.6)	<0.001
Smoking, *n* (%)			
Current	884 (21.2)	526 (13.7)	<0.001
Former	1,092 (26.2)	1,233 (32.1)	
Never	2,188 (52.5)	2,081 (54.2)	
Overweight, *n* (%)	1,690 (40.4)	1,550 (40.2)	<0.001
Obesity, *n* (%)	946 (22.6)	1,766 (45.9)	
Physical activity, *n* (%)			
None	1,018 (24.3)	1,346 (34.9)	<0.001
Low to moderate	1,765 (42.1)	1,646 (42.6)	
Moderate	995 (23.7)	723 (18.7)	
Vigorous	412 (9.8)	146 (3.8)	
Hyperuricemia, *n* (%)	354 (10.0)	772 (22.9)	<0.001
SCORE			
Low risk	821 (30.1)	166 (5.9)	
Moderate risk	1,059 (38.8)	732 (26.1)	<0.001
High risk	326 (12.0)	539 (19.2)	
Very high risk	520 (19.1)	1,367 (48.8)	
Hypertension-mediated organ damage			
Pulse pressure >60 mmHg (≥65 years), *n* (%)	298 (7.1)	1,050 (27.2)	<0.001
Ankle-brachial index <0.9, *n* (%)	53 (1.3)	82 (2.1)	<0.01
Left ventricular hypertrophy, *n* (%)	57 (1.4)	260 (6.7)	<0.001
Urinary albumin excretion, *n* (%)			
<30 mg/g	4,007 (95.6)	3,428 (88.8)	<0.001
30–299 mg/g	172 (4.1)	386 (10)	
≥300 mg/g	11 (0.3)	46 (1.2)	
Moderate eGFR >30–59 ml/min/1.73 m^2^	107 (2.6)	470 (12.3)	<0.001
Any hypertension-mediated organ damage^a^, *n* (%)	604 (14.7)	1,695 (44.3)	<0.001
Vascular disease			
Ischemic heart disease, *n* (%)	154 (3.7)	429 (11.1)	<0.001
Stroke, *n* (%)	88 (2.1)	235 (6.1)	<0.001
Peripheral artery disease, *n* (%)	133 (3.2)	267 (6.9)	<0.001
Heart failure, *n* (%)	49 (1.2)	200 (5.2)	<0.001
Retinopathy, *n* (%)	15 (0.4)	32 (0.8)	0.296
Chronic kidney disease, *n* (%)			
MDRD	190 (4.6)	601 (15.7)	<0.001
CKD-EPI	142 (3.4)	532 (13.9)	<0.001
Atrial fibrillation, *n* (%)	112 (2.7)	353 (9.1)	<0.001
Cardiovascular disease^b^, (%)	391 (9.3)	931 (24.1)	<0.001

Hyperuricemia, *n* (%) >7 men; >6 women, eGFR, estimated glomerular filtration rate.

^a^ Includes pulse pressure >60 mmHg, ankle-brachial index <0.9; left ventricular hypertrophy, albuminuria ≥30 mg/g or eGFR, estimated glomerular filtration rate >30–59 ml/min/1.73 m^2^.

^b^ Includes stroke, ischemic heart disease, retinopathy, heart failure or peripheral artery disease.

The risk of having hypertension increased with the presence of associated CVRF and comorbidities, particularly diabetes and obesity. By contrast, the risk of hypertension decreased with the intensity of physical activity. Similarly, the risk of hypertension increased with the presence of increased urinary albumin excretion, ischemic heart disease, stroke, peripheral artery disease, heart failure, chronic kidney disease and atrial fibrillation. This was particularly high in patients with chronic kidney disease, atrial fibrillation, urinary albumin excretion ≥300 mg/g, and stroke (Table 3).

**Table 3:** Risk of hypertension according to the association of cardiovascular risk factors, hypertension-mediated organ damage and cardiovascular disease.

	OR	95% confidence interval	*p*
Cardiovascular risk factors			
Age	1.07	1.06–1.07	<0.001
Woman	1.40	1.24–1.57	<0.001
Hypercholesterolemia	1.95	1.76–2.16	<0.001
Diabetes	2.69	2.35–3.08	<0.001
Smoking			
Current	Reference	Reference	
Former	1.08	0.93–1.26	0.305
Never	0.92	0.80–1.06	0.260
Overweight			
BMI 25–26.99 kg/m^2^	1.99	0.86–4.59	0.109
BMI 27–29.99 kg/m^2^	2.79	1.21–6.43	0.016
Obesity			
BMI 30–34.99 kg/m^2^	4.97	2.15–11.45	<0.001
BMI 35–39.99 kg/m^2^	6.67	2.85–15.61	<0.001
BMI 40–49.99 kg/m^2^	8.83	3.61–21.58	<0.001
BMI ≥50 kg/m^2^	8.52	2.17–33.42	0.002
Physical activity			
None	Reference	Reference	
Low–moderate	0.68	0.61–0.77	<0.001
Moderate	0.60	0.52–0.69	<0.001
Vigorous	0.50	0.40–0.63	<0.001
Metabolic syndrome	4.28	3.84–4.76	<0.001
Hyperuricemia	2.36	2.03–2.75	<0.001
Excessive alcohol consumption	1.25	1.08–1.45	0.003
SCORE			
Low risk	Reference	Reference	
Moderate risk	1.81	1.47–2.22	<0.001
High risk	3.19	2.51–4.07	<0.001
Very high risk	4.29	3.40–5.40	<0.001
Hypertension-mediated organ damage			
Urinary albumin excretion			
<30 mg/g	Reference	Reference	
30–299 mg/g	1.89	1.53–2.33	<0.001
≥300 mg/g	3.13	1.51–6.51	0.002
Vascular disease			
Ischemic heart disease	2.27	1.84–2.80	<0.001
Stroke	3.02	2.36–3.88	<0.001
Peripheral artery disease	2.27	1.83–2.80	<0.001
Heart failure	2.28	1.60–3.24	<0.001
Retinopathy	1.43	0.73–2.79	0.296
Chronic kidney disease (CKD EPI)	4.53	3.75–5.49	<0.001
Atrial fibrillation	3.66	2.95–4.55	<0.001
CV disease^a^	3.09	2.72–3.51	<0.001

OR, odds ratio; BMI, body mass index; SCORE, cardiovascular risk table.

^a^ Includes stroke, ischemic heart disease, retinopathy, heart failure or peripheral artery disease.

Antihypertensive treatment in the whole hypertensive population and according to gender is shown in Table 4. Overall, 58.8% of patients followed the recommendations about diet and physical activity. For different reasons 6.1% of patients did not take any drug, 38.8% were taking one prescribed antihypertensive drug, 35.5% two antihypertensive drugs and the remaining 19.6% three or more prescribed drugs. The most commonly prescribed antihypertensive agents were renin-angiotensin-system (RAS) blockers 81% (ACEi 38.5%, ARB 42.5%) followed by diuretics 45.3%, particularly thiazide or thiazide-like diuretics. Diuretics were taken more frequently by women (41.6% vs. 49.0%: *p* < 0.001), whereas ACEi (41.8% vs. 35.1%; *p* < 0.001) and alpha blockers (4.8% vs. 1.4%; *p* < 0.001) were more frequent in men. Treatment with statins was higher in patients with hypertension compared with those without hypertension (76.7% vs. 56.6%, *p* < 0.001).

**Table 4:** Antihypertensive treatment in patients with hypertension.

	Men (*n* = 1,946; 50.5%)	Women (*n* = 1,914; 49.5%)	Total (*n* = 3,860; 100%)	*p*
Diet and physical activity, *n* (%)	1,165 (59.8)	1,106 (57.8)	2,271 (58.8)	0.309
Diuretic, *n* (%)	810 (41.6)	938 (49.0)	1,748 (45.3)	<0.001
Loop diuretic, *n* (%)	106 (5.4)	120 (6.3)	226 (5.9)	0.725
Thiazide like diuretic, *n* (%)	542 (27.8)	633 (33.1)	1,175 (30.4)	0.001
Potassium sparing diuretic, *n* (%)	54 (2.8)	57 (3.0)	111 (2.9)	0.998
Angiotensin receptor blocker, *n* (%)	820 (42.1)	820 (42.9)	1,640 (42.5)	0.851
Angiotensin-converting enzyme inhibitor, *n* (%)	815 (41.8)	671 (35.1)	1,486 (38.5)	<0.001
Calcium channel blocker, *n* (%)	475 (24.4)	406 (21.2)	881 (22.8)	0.008
Beta blocker, *n* (%)	386 (19.8)	349 (18.2)	735 (19.0)	0.084
Alpha blocker, *n* (%)	94 (4.8)	26 (1.4)	120 (3.1)	<0.001
Renin inhibitor, *n* (%)	14 (0.7)	12 (0.6)	26 (0.7)	0.764
Central acting agents, *n* (%)	0	1 (0.05)	1 (0.02)	0.998
Others, *n* (%)	26 (1.3)	15 (0.8)	41 (1.1)	0.091
Number of antihypertensive drugs, *n* (%)				
0	114 (5.9)	122 (6.4)	236 (6.1)	0.003
1	753 (38.7)	743 (38.8)	1,496 (38.8)	
2	658 (33.8)	713 (37.3)	1,371 (35.5)	
3	333 (17.1)	277 (14.5)	610 (15.8)	
4	73 (3.8)	54 (2.8)	127 (3.3)	
5	14 (0.7)	5 (0.3)	19 (0.5)	
6	1 (0.1)	0	1 (0.0)	

**Figure 1:**
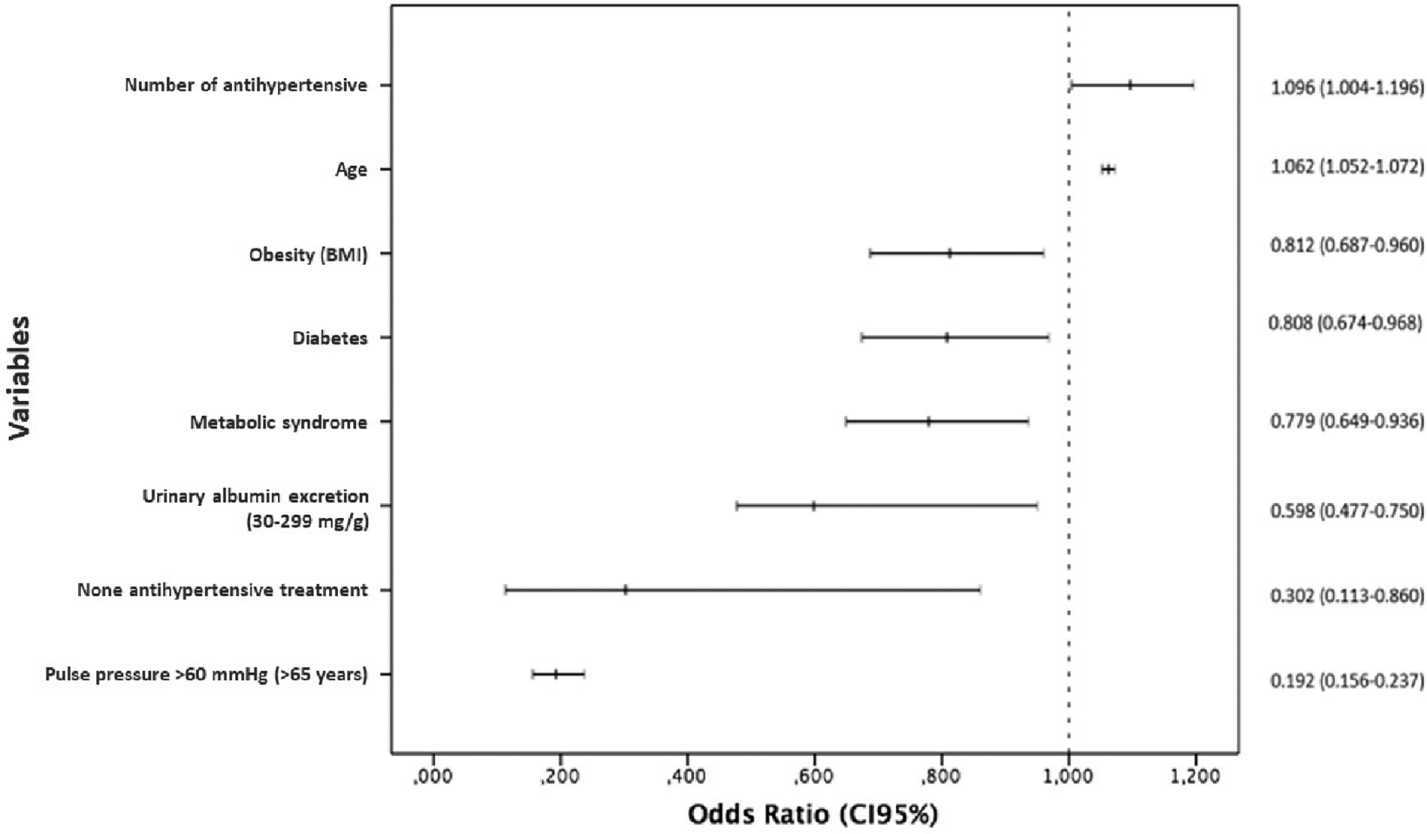
Variables associated to blood pressure control in hypertensive patients*. *Multivariate logistic regression, stepwise backward method (LR). CI, Confidence interval; Number of antihypertensive drugs; Age; Obesity; Diabetes; Metabolic syndrome; Urinary albumin excretion (30–299 mg/g); None antihypertensive treatment; Pulse pressure >60 mmHg (≥65 years).

Overall, 58.3% of hypertensive patients achieved recommended BP targets. There was a trend towards a higher BP control among women than men (61.6% vs. 57.8%; *p* = 0.036). According to the binary logistic regression model, the variables which were likely to associate independently with BP control were male/female gender, age, diabetes, obesity, the metabolic syndrome, current smoking, urinary albumin excretion (30–299 mg/g), pulse pressure >60 mmHg (≥65 years), any HMOD, retinopathy, antihypertensive treatment and number of antihypertensive drugs (Table 5). Figure 1 shows the variables resulting from the final model. The strongest independent positive association with BP control was observed in relation to the number of antihypertensive drugs (OR: 1.10; *p* < 0.041), where the higher the number of drugs the better BP control, and with older age (OR: 1.06; *p* < 0.001). A negative relationship with BP control was found with diabetes, obesity, the metabolic syndrome, urinary albumin excretion, high pulse pressure, and lack of antihypertensive treatment.

**Table 5:** Blood pressure control in relation to the association of cardiovascular risk factors, hypertension-mediated organ damage, clinical cardiovascular disease, and antihypertensive treatment.

	Blood pressure control: No (*n* = 1,610; 41.7%)	Blood pressure control: Yes (*n* = 2,250; 58.3%)	*p*
Men, *n* (%)	650 (42.2)	892 (57.8)	0.036
Woman, *n* (%)	581 (38.4)	931 (61.6)	
Age, years, *n* (%)			
18–50	157 (52.2)	144 (47.8)	<0.001
51–64	447 (45.3)	539 (54.7)	
65–79	545 (38.4)	874 (61.6)	
≥80	82 (23.6)	266 (76.4)	
Hypercholesterolemia, *n* (%)	810 (39.6)	1,233 (60.4)	0.290
Diabetes, *n* (%)	464 (45.4)	558 (54.6)	<0.001
Obesity (BMI), *n* (%)	618 (44.0)	788 (56.0)	<0.001
Current smoking, *n* (%)	182 (46.7)	208 (53.3)	0.006
Metabolic syndrome, *n* (%)	783 (43.9)	1,001 (56.1)	<0.001
SCORE, *n* (%)			
Low risk	97 (36.2)	171 (63.8)	0.017
Moderate risk	275 (37.5)	458 (62.5)	
High risk	289 (39.0)	452 (61.0)	
Very high risk	570 (43.4)	742 (56.6)	
Hyperuricemia, *n* (%)	261 (41.4)	370 (58.6)	0.537
Left ventricular hypertrophy, *n* (%)	94 (46.1)	110 (53.9)	0.082
Ankle-brachial index <0.9, *n* (%)	36 (40.0)	54 (60.0)	0.952
Urinary albumin excretion (30–299 mg/g), *n* (%)	221 (51.2)	211 (48.8)	<0.001
Pulse pressure >60 mmHg (≥65 years), *n* (%)	472 (55.3)	381 (44.7)	<0.001
eGFR >30–59 ml/min/1.73 m^2^, *n* (%)	152 (39.7)	231 (60.3)	0.769
Any hypertension-mediated organ damage^a^, *n* (%)	648 (50.5)	635 (49.5)	<0.001
Ischemic heart disease, *n* (%)	120 (36.4)	210 (63.6)	0.122
Stroke, *n* (%)	69 (36.9)	118 (63.1)	0.327
Heart failure, *n* (%)	62 (39.5)	95 (60.5)	0.830
Peripheral arterial disease, *n* (%)	60 (40.8)	87 (59.2)	0.897
Atrial fibrillation, *n* (%)	96 (38.6)	153 (61.4)	0.556
Retinopathy, *n* (%)	17 (60.7)	11 (39.3)	0.027
Chronic kidney disease (CKD EPI), *n* (%)	169 (38.9)	265 (61.1)	0.511
Cardiovascular disease^b^, *n* (%)	283 (38.2)	458 (61.8)	0.177
Antihypertensive treatment, *n* (%)	1,214 (40.1)	1,815 (59.9)	0.005
Number of antihypertensive drugs, *n* (%)			
0	102 (56.4)	79 (43.6)	<0.001
1	450 (39.0)	704 (61.0)	
2	426 (38.8)	673 (61.2)	
≥3	253 (40.8)	367 (59.2)	

Hyperuricemia, *n* (%) >7 men; >6 women, eGFR, estimated glomerular filtration rate; SCORE, cardiovascular risk table; BMI, body mass index.

^a^ Includes pulse pressure >60 mmHg, ankle-brachial index <0.9; left ventricular hypertrophy, albuminuria ≥30 mg/g or eGFR: estimated glomerular filtration rate >30–59 ml/min/1.73 m^2^.

^b^ Includes stroke, ischemic heart disease, retinopathy, heart failure or peripheral artery disease.

## Discussion

This study performed in a wide sample of patients recruited by general practitioners in Spain showed that approximately half of patients attended in primary care setting had hypertension. Patients with hypertension had a worse clinical profile than non-hypertensive patients, with more associated CVRF, HMOD and clinical CVD. Although the majority of this patients were treated with combinations of antihypertensive drugs, approximately four out of ten patients did not achieve the recommended BP goals.

The IBERICAN study shows a real map of the burden of CVRF and CVD in daily clinical practice in primary care centers in Spain. In the IBERICAN cohort, 48% of patients had hypertension. This frequency was higher than that reported in population-based surveys (30%–35%) (4, 5, 9, 10, 22). However, it should be noted that IBERICAN was a clinical-based study of patients seen in primary care setting. IBERICAN is a very useful study that will help to increase knowledge about CV risk in primary care settings (23) and these data clearly indicate that hypertension is the major health care problem that should be ruled out in every patient without a previous history of hypertension attended by general practitioners (4).

As expected, patients with hypertension had more associated CVRF, HMOD and clinical CVD, confirming previous results by other authors (9–14). The risk of having hypertension is associated with other CVRF as diabetes, obesity, and the metabolic syndrome, that increased the risk, or intensity of physical activity, that reduced it. Our results confirm the impact of excess weight, salt intake and low physical activity that cause close of 50% of the hypertension cases (7). This relationship highlighted the importance to a correct management of all CVRF, including promotion of a healthy lifestyle, and not only hypertension, to reduce the global cardiovascular risk of the patients (24, 25). Our results also showed an important relationship of hypertension risk development with epidemiological data as male gender, age over 65 years, be pensioned, living in a rural area, lower educational level, and low income. These results completed the role described by other authors about the social determinants in the development of hypertension and BP control (12, 26).

Approximately 60% of hypertensive patients of the IBERICAN cohort achieved the BP targets recommended by the guidelines. With regard to treatment, 6.1% of patients did not take any drug, 38.8% were taking one prescribed antihypertensive drug, 35% two antihypertensive drugs and 20% three or more prescribed drugs. Our results show the trends in Spain, where we observed an increase in BP control from 36% in 2002 to 46% in 2010 among hypertensive patients treated by primary care physicians (27), mainly related with a progressive higher use of combination therapy (from 44% to 64%) (27), similarly to other studies describing a direct relationship between the use of combination treatment and improvement of BP control (60%–65%) (13, 28). Even considering a great variability among different countries (4), BP control rates have remained stable in the last decade in the world (30%–50%), despite the improvement in detection, awareness, treatment and control of BP in high-income countries along the 80s and 90s (5). The challenge of improving BP control worldwide may be overcome, and our results clearly indicate how other associated CVRF and the increase in the use of antihypertensive combination strategies could improve this control, addressing the main clinical problems of therapeutic inertia (29) and adherence to chronic cardiovascular treatments (30).

Interestingly, in contrast with previous reports (31) our results show that patients with better BP control had more frequently diabetes, obesity, hypercholesterolemia, current smoking, LOMH and CVD compared to uncontrolled patients, exhibiting a higher CV risk. A probable explanation is that the identification of these conditions by primary care physicians let them to a better stratification of the patient global CV risk and intensify pharmacological treatment. It would be very interesting what will be the effect of this situation in the prognosis of patients of our cohort when we can analyze the cardiovascular risk in the follow up along the next years.

This study has some limitations. The participation of primary care physicians was volunteer and not random, which does not allow to strictly generalize our results to the population daily attended in primary care. Besides, the number of included patients per physician and year was very low, what may be a potential major bias in the interpretation of the results. It may be speculated that only physicians most interested in CVD were those participating as researchers. This could explain the excellent reported data concerning antihypertensive drug prescription and BP control, what may not be extrapolated to real clinical practice.

Our aim was to analyze the relationship between the CVRF with the prevalence of hypertension and BP control with other variables, and the sample recruited let us to make these analyses with a high statistical power. In addition, when comparing frequencies, the lack of control group only permits to perform indirect comparisons with the results obtained in other studies. In fact, the results of this study probably represent the best and more actually scenario in hypertension control in the population cared by general practitioners of the Public Health System in Spain, and perhaps the situation, specially in the BP control, is worst in real world. In any case, this bias does not invalidate the observed association among hypertension and other CVRF, HMOD, and CVD in our study.

In conclusion, patients with hypertension are very common in the primary care setting, exhibiting a worse clinical profile that deserves a more aggressive and comprehensive approach to actually reduce CV burden. Despite that four out of ten patients do not achieve recommended BP goals many of them are still treated in monotherapy. It is necessary to develop strategies to implement a more appropriate and intense treatment in hypertensive patients through the use of combination therapy, preferably in a single pill fixed-dose combination to improve medication adherence, long-term persistence, and CV morbidity and mortality.

## Data Availability

The original contributions presented in the study are included in the article/**Supplementary Material**, further inquiries can be directed to the corresponding author.
